# Study on the Transverse Properties of T800-Grade Unidirectional Carbon Fiber-Reinforced Polymers

**DOI:** 10.3390/ma18040816

**Published:** 2025-02-13

**Authors:** Hao Wang, Xiang-Yu Zhong, He Jia, Lian-Wang Zhang, Han-Song Liu, Ming-Chen Sun, Tian-Wei Liu, Jian-Wen Bao, Jiang-Bo Bai, Si-Cheng Ge

**Affiliations:** 1 Beijing Institute of Space Mechanics & Electricity, Beijing 100190, China; wanghao_9295@163.com (H.W.);; 2 School of Transportation Science and Engineering, Beihang University, Beijing 100191, China; 3 Jingdezhen Research Institute, Beihang University, Jingdezhen 333000, China; 4 National Key Laboratory of Advanced Composite, AVIC Composite Technology Center, AVIC Composite Corporation Ltd., Beijing 101300, Chinaliuhansongzhfc@foxmail.com (H.-S.L.); baojianwen@hotmail.com (J.-W.B.); 5 School of Automation Science and Electrical Engineering, Beihang University, Beijing 100191, China; liutianwei@buaa.edu.cn

**Keywords:** T800-grade unidirectional carbon fiber-reinforced polymers, transverse tension and compression, fractography experiments, micromechanical finite element method

## Abstract

This paper focuses on the transverse tensile and compressive mechanical properties of T800-grade unidirectional (UD) carbon fiber-reinforced polymers (CFRPs). Firstly, transverse tensile and compressive tests were conducted on UD composite laminates, yielding corresponding stress–strain curves. The results indicated that, for tension, the transverse tensile modulus, strength, and failure strain were 8.7 GPa, 64 MPa, and 0.74%, respectively, whereas for compression, these values were 8.4 GPa, 197.1 MPa, and 3.43%, respectively. The experimental curves indicated brittle failure under tensile loadings and significant plastic failure characteristics under compressive loading for the T800-grade composite. Subsequently, fractography experiments were performed to observe the fracture morphologies, revealing that tensile fractures were through-thickness cracks perpendicular to the loading direction, while compressive fractures were at a 52° angle to the loading direction. Finally, a micromechanical finite element method (FEM) was employed to simulate the tensile and compressive failure processes of the unidirectional composite, and the tensile and compressive properties were predicted. The simulation results showed that under both tensile and compressive loadings, interfacial elements failed first, causing stress concentration and damage to nearby resin elements. The damaged resin and interfacial elements expanded and connected, leading to ultimate failure. The predicted tensile stress–strain curve exhibited linear characteristics consistent with the experimental results in most regions but showed more pronounced nonlinearity before ultimate failure. The predicted compressive stress–strain curve aligned well with the experimental results in terms of nonlinearity. The predicted elastic modulus, failure strengths, and failure strains were in good agreement with the experimental results, with differences of 1.1% (tension modulus), 3.2% (tension strength), and 13.5% (tension failure strain), and 3.6% (compression modulus), −8.5% (compression strength), and −3.8% (compression failure strain). The final failure morphologies were in good accordance with the fractography experimental observations.

## 1. Introduction

Carbon fiber, with its high specific strength and modulus, fatigue resistance, and low thermal expansion coefficient, is widely used in aviation, aerospace, machinery, and other fields for the preparation of high-performance polymer-based composites reinforced with it [1]. The first generation of high-performance carbon fiber technology, represented by T300 and AS4, emerged in the 1960s to 1970s, primarily being used for lower-stress structural components in aviation, such as aircraft spoilers, rubbers, and fairings [2,3]. To further lighten aircrafts, the second generation, represented by T800 and IM7, was developed in the 1980s, featuring strengths over 5.5 GPa and moduli between 290 and 300 GPa. Compared to T300, T800-grade carbon fiber boasts a relatively smaller single-filament diameter, accompanied by a more compact and orderly crystal structure, as well as a higher degree of graphitization. These attributes collectively contribute to its superior tensile strength, modulus, thermal stability, and chemical resistance [4,5]. As a result, T800 carbon fiber has found extensive application in critical load-bearing aircraft components. For instance, Boeing’s B787 primary fuselage and wing structures use T800/3900-2 high-toughness epoxy composites [6], while Airbus’s A350 center wing box and wings employ Hexcel’s IMA/M21 high-toughness epoxy composites, with composite structures accounting for over 50% [7].

The foundation for applying advanced carbon fiber-reinforced polymers (CFRPs) in engineering lies in obtaining accurate mechanical properties of unidirectional (UD) composites. The most direct and versatile method is experimental testing, with established standards such as ASTM and EN series, which can directly measure mechanical properties like the strength and stiffness of unidirectional composites with accurate and reliable results. However, this method consumes significant materials and equipment resources, and the results obtained for a given UD CFRP cannot be directly extrapolated to other systems with different fiber volume fractions or constituent characteristics, leading to relatively high costs for studying its mechanical properties. The analytical method involves establishing mathematical models and formulas to predict and calculate the mechanical properties of unidirectional composites [8,9,10]. This method can overcome the limitations of experimental methods and is suitable for predicting the mechanical properties of unidirectional composites under different design requirements and molding processes. However, its accuracy is affected by mathematical models and formulas, and there are still challenges in characterizing the evolution of in situ damage. With the rapid development of computer technology and the finite element method, computational micromechanics has gradually become an effective tool for understanding composite damage processes and predicting composite properties. This method has high accuracy in predicting the mechanical behavior of unidirectional composites and can clearly reveal the damage mechanisms of unidirectional composites under different loading conditions [11,12,13].

The transverse mechanical properties of unidirectional composites are crucial, providing necessary stiffness and strength for composite structures under multi-axial loading conditions. Transverse fracture often occurs at the initial stage of loading and is a part of the ultimate design criteria for composite structures. Compared to longitudinal failure, transverse failure of composites is more complex, mainly dominated by interface and matrix damage, with more uncertainties in experimental characterization and less related experimental data [14]. The micromechanical finite element method (FEM) is an effective tool for describing the damage process of unidirectional composites under transverse loading and can accurately predict their mechanical properties. Gonzalez et al. [14] were among the first to use the micromechanical FEM to study the microscopic failure mechanism of UD CFRPs under transverse compression, constructing a 2D micromechanical representative volume element (RVE) finite element model with a random fiber distribution and applying periodic boundary conditions to ensure the continuity of boundary loadings and displacements. Elastoplastics based on the Mohr–Coulomb criterion and bilinear constitutive models were used to simulate the material models of the resin and interface, respectively. The transverse compressive stress–strain curve of the composite was obtained through simulation, and a parametric study was conducted to investigate the influence of interface strength properties on the stress–strain curve. Building on this, Yang et al. [15] studied the micromechanical failure mechanism of unidirectional composites under transverse tension and compression, simulating the initiation and evolution of internal damage, which showed consistent micromechanical failure characteristics with experimental results. Additionally, parametric analyses were conducted to investigate the effects of interface stiffness, fracture energy, and interface strength on the stress–strain response and failure process. In addition, numerous research efforts have been dedicated to refining and expanding micromechanical finite element models. In terms of RVE models, 3D micromechanical models [16,17] and extended interface models [16] have been developed; for resin material models, elastoplastic models based on the extended linear Drucker–Prager criterion [15], thermo-acoustic elastoplastic models [17], elastoplastic models with damage based on the ellipse–parabola criterion [18], and modified Drucker–Prager elastoplastic models [19] have been proposed; and for interface material models, the cohesive friction damage model [20] has been introduced. It can be seen that there are extensive studies on the transverse mechanical properties of carbon fiber-reinforced resin-based composites. However, composites are composed of multiphase materials, and different reinforcement/polymer matrix systems often exhibit different mechanical behavior characteristics. The composite systems studied in the aforementioned research include AS4/8552 [14,15,20], AKSACA/epoxy [16,18], and IM7/8552 [19], most of which focus on T300-grade CFRPs, while there is relatively little research on the transverse mechanical properties of T800-grade CFRPs.

In summary, T800-grade fibers, due to their higher carbon content and better microstructure, exhibit superior mechanical properties compared to T300-grade fibers, leading to their broader application in scenarios requiring high performance. However, despite their advantages, there has been relatively limited research focusing on the mechanical properties of composites made from T800-grade fibers; comprehensive studies are particularly lacking. In a previous study [21], the author employed experimental and micromechanical FEMs to investigate the mechanical behavior of a T800-grade UD CFRP under longitudinal tensile loading. To comprehensively deepen the understanding of the mechanical properties of this composite system, it is necessary to further systematically study its transverse mechanical properties through experiments and a micromechanical FEM, revealing its mechanical characteristics and failure mechanisms under transverse loading. This will lay the foundation for subsequent engineering applications.

The structure of this paper is as follows: Firstly, the transverse tensile and compressive properties of a T800-grade UD CFRP were tested, and the corresponding stress–strain curves were obtained. Fractography experiments were also conducted on the fracture surfaces of the specimens. Then, a micromechanical damage model considering the random spatial distribution of fibers was established using the finite element method to simulate the damage behavior of the composites under transverse tensile and compressive loading. The stress–strain curves obtained through calculations were combined with simulation results to analyze the damage initiation, damage evolution, and ultimate failure processes of the composites under transverse tensile and compressive loading. By comparing the simulation results with experimental tests and fracture analysis results, the validity of the numerical method was verified. This study systematically investigates the transverse mechanical properties of a T800-grade UD CFRP through experimental and micromechanical finite element methods, and explores the underlying failure mechanisms. The results obtained from this study offer valuable insights for the optimal design and engineering applications of such high-performance carbon fiber-reinforced polymer composites.

## 2. Mechanical Property Testing

### 2.1. Materials and Specimens

T800-grade carbon fiber, with a tow specification of 12K and a density of 1.81 g/cm^3^, was processed into a carbon fiber-reinforced epoxy prepreg through a hot-melt method. The single-layer thickness of the prepreg was 0.14 mm.

The T800-grade UD CFRP laminate used for transverse tensile testing was made of 16 prepreg layers through autoclave processing. The laminate stacking sequence was [90]_16_, with a thickness of 2.32 mm ± 0.04 mm. The specimens for transverse tensile testing were cut from the laminate plates by a digital cutting machine with a nominal dimension of 250 mm × 15 mm, as described in the test standard [22]. The 56 mm regions at both ends of the specimen were reinforced with glass fiber tabs to prevent stress concentration in the grip sections from affecting the failure area. The glass fiber tabs were adhered to the test specimens using a high-strength epoxy adhesive. In view of the test standard [22], which requires five or more test pieces to ensure the availability of valid data, six specimens were tested in this paper.

The T800-grade UD CFRP laminate used for transverse compressive testing is the same laminate stacking as that for tensile testing, with a thickness of 2.35 mm ± 0.04 mm. The nominal dimensions of the transverse compressive specimens were 140 mm × 13 mm, as depicted in the test standard [23]. In the center, a 13 mm working area was preserved, while the surrounding area was reinforced with tabs using a high-strength epoxy adhesive to prevent stress concentration in the chuck section from affecting the failure area. As with the tensile specimens, six compression specimens underwent testing to ensure that valid test data were obtained.

### 2.2. Transverse Tensile Test

**Figure 1:**
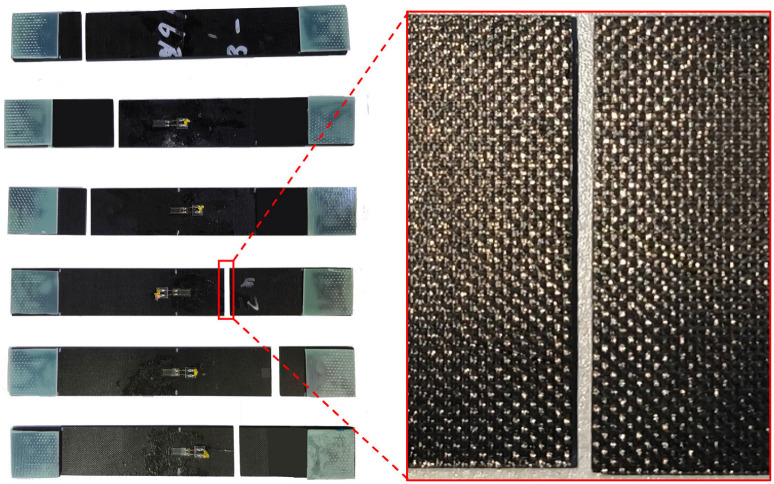
Image of T800-grade UD CFRP specimens after transverse tensile failure.

In accordance with the ASTM D3039 standard [22], transverse tensile tests were conducted on specimens, utilizing tensile clamps on the MTS E45 electronic universal testing machine, manufactured by MTS Systems Corporation in Shanghai, China, equipped with a load cell that had a capacity of 300 kN. To ensure quasi-static loading, the test speed was set to 2.5 mm/min with a sampling frequency of 20 Hz. Strain gauges, specifically the BE120-5AA-P500 type, produced by China Aerospace Science and Industry Corporation Electric Measurement Instruments Co., Ltd. (Beijing, China), were securely adhered to the specimen surfaces using 502 glues, and strategically positioned to capture critical strain data, in conjunction with the JM3840 dynamic and static strain measurement system from Yangzhou Jingming Technology Corporation in Yangzhou, China. The states of the tensile specimens after failure are shown in Figure 1. The fracture locations are all within the working section, indicating the validity of the test data.

### 2.3. Transverse Compressive Test

**Figure 2:**
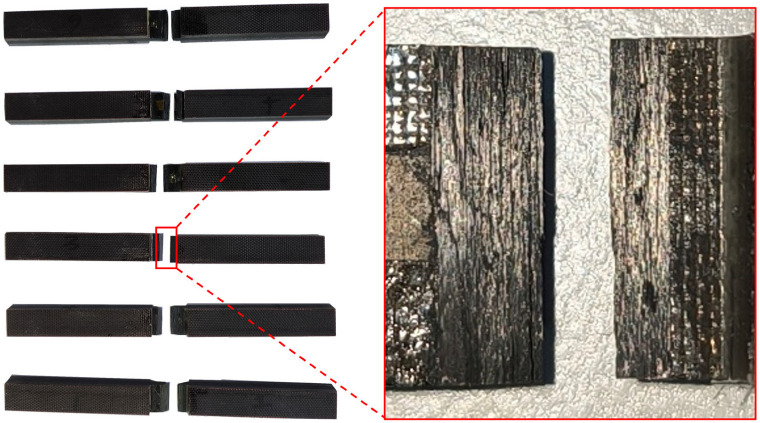
Image of T800-grade UD CFRP specimens after transverse compressive failure.

Based on the ASTM 6641 standard [23], transverse compressive tests were performed on specimens using compressive clamps on the same MTS E45 machine. To maintain a quasi-static loading condition, the test was conducted at a speed of 2.5 mm/min, with a sampling frequency of 20 Hz. For strain data acquisition, strain data were captured using strain gauges with the aforementioned JM3840 strain measurement system. The conditions of the specimens after failure are presented in Figure 2, respectively, with all fracture sites occurring within the working segment. This confirms the reliability of the test data obtained.

### 2.4. Fractography Experiments

**Figure 3:**
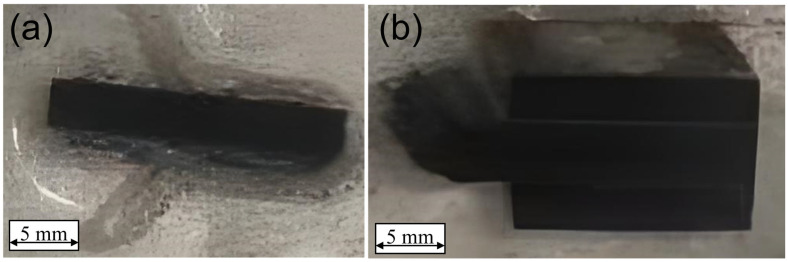
Fractography observation experiment on the T800-grade UD CFRP: (**a**) typical transverse tensile specimen; (**b**) typical transverse compressive specimen.

The fracture surfaces of the T800-grade UD CFRP specimens subjected to transverse tension and compression were cut and cast into round fractography samples. The typical transverse tensile and compressive specimens are shown in Figure 3a and Figure 3b, respectively. The fracture specimens have a length of approximately 20 mm, with widths measuring 10 mm, and the round mounts have a diameter of 50 mm. These samples were then ground and polished using the MECATHCH 250 DPC grinding–polishing machine, manufactured by PRESI in Contamine-sur-Arve, France. A ZEISS Smartzoom 5 digital optical microscope, manufactured by Carl Zeiss in Oberkochen, Germany, was employed to observe the samples perpendicular to the fiber direction.

## 3. Construction of the Computational Model

### 3.1. Micromechanical Finite Element Model

**Figure 4:**
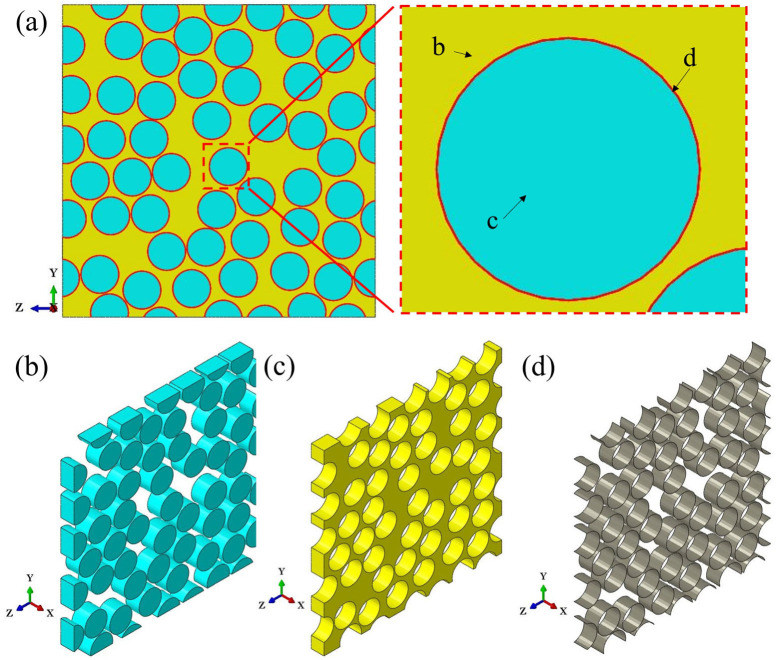
Schematic diagram of finite element models for predicting the transverse properties of composite materials: (**a**) RVE; (**b**) fibers; (**c**) matrix; (**d**) interfaces.

**Figure 5:**
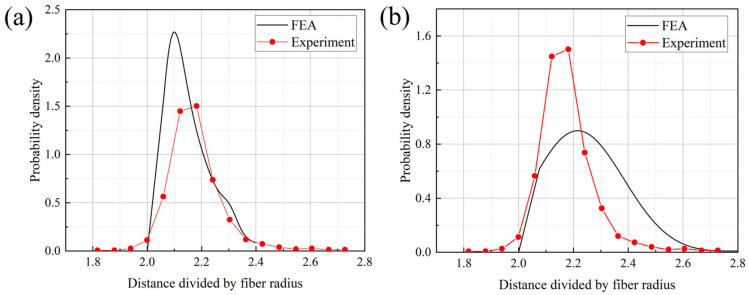
Statistical results of the fiber spatial distribution and experiment [25]: (**a**) nearest neighbor distribution function; (**b**) second nearest neighbor distribution function.

**Figure 6:**
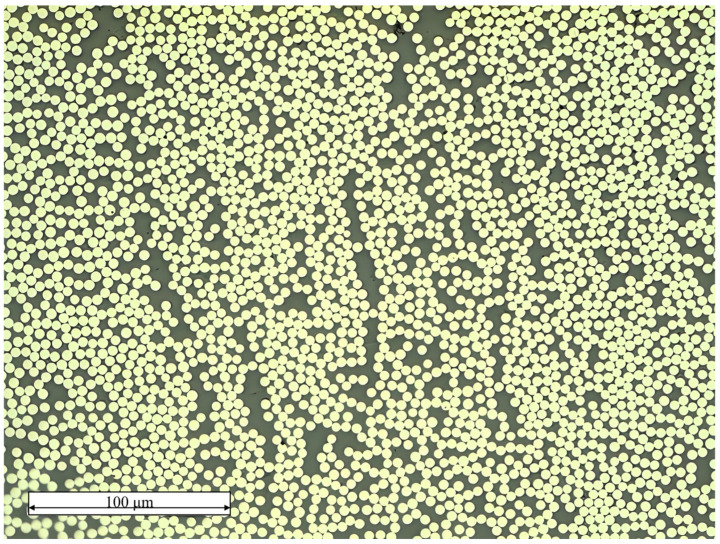
Sample image chosen to obtain the volume fraction and diameter of T800-grade fibers.

The primary approach in employing micromechanics to characterize the macroscopic mechanical properties of composites involves utilizing a unit cell (UC) or representative volume element (RVE) to represent the material’s mechanical behavior on an infinite (macro) scale based on a limited micromechanical scale [24]. The initial step in predicting the mechanical properties of composites is to construct an RVE that adequately exhibits equivalent mechanical properties. When predicting the transverse mechanical properties of UD composites using micromechanics, an RVE containing randomly spatially distributed fibers is typically constructed, i.e., cylindrical fibers randomly distributed within a cubic volume. It is taken for granted that the larger the scale of the RVE, the more accurately it can reflect the mechanical behavior of the macroscopic material. However, computational cost also needs to be taken into consideration. The RVE model containing 50 fibers in its cross-section was found to be sufficient for predicting the transverse mechanical properties and capturing the failure characteristics of the composites [12,19]. Therefore, in this paper, a finite element RVE model containing 50 fibers, along with the surrounding interface and resin elements, is constructed, as shown in Figure 4a. The random distribution of 50 fibers in space is realized according to the random sequential expansion method (RSM) [25], as shown in Figure 4b. The representativeness of the fiber spatial distribution is demonstrated through statistical results, as illustrated in Figure 5a and Figure 5b, respectively. The results show that the peaks of the probability density curves for the nearest distance and the second nearest distance between fiber centers are close to those obtained from experimental results. The discrepancies in the remaining portions are mainly due to the fact that the fiber distribution in real composites is generally not completely random [25]. Additionally, to facilitate the application of boundary conditions, the boundaries of the RVE model are periodic, meaning that the fiber models at the boundaries are incomplete and are completed by joining with models at the opposite boundaries to form whole fibers. The resin model, shown in Figure 4c, is distributed around the fiber elements. The interface model is assumed to be a ring wrapping around the fiber elements, with a thickness of 0.1 μm, as illustrated in Figure 4d. This thickness value is based on the research conducted by Chen et al. [26]. The volume fraction and diameter of the fibers in the T800-grade UD CFRP were obtained through the method described in [27], combined with microscopic images from fractography experiments as shown in Figure 6. The average volume fraction is 58% and the average fiber diameter of the fibers is 5.1 μm, resulting in an RVE longitudinal cross-sectional size of 42 μm × 42 μm. Assuming that the fibers are all arranged longitudinally and completely aligned, Garoz et al. [28] studied the influence of RVE longitudinal size on the prediction of transverse mechanical properties and found that thickness had no significant effect on the prediction results. Therefore, the thickness of the RVE model in this paper is set to 3 μm, considering numerical computation efficiency.

**Figure 7:**
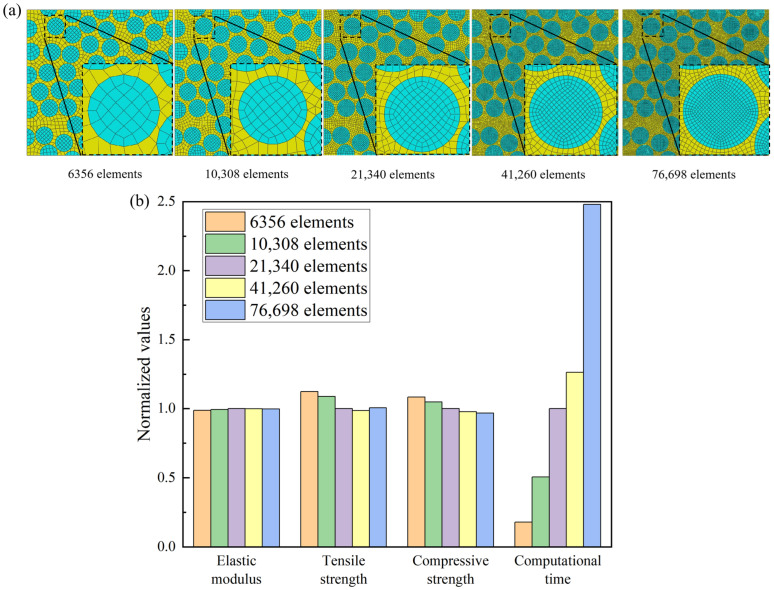
Mesh convergence analysis: (**a**) five RVEs with different numbers of elements; (**b**) normalized predictions of mechanical properties and computational times for five RVEs.

Through mesh convergence analysis, the number of meshes for the finite element model of the RVE was determined. Five RVEs with varying mesh quantities, ranging from 6356 to 76,698 elements, were constructed in the finite element software ABAQUS 2017, as shown in Figure 7a. Figure 7b presents the normalized results of the mechanical property calculations and computational time consumption for the five RVEs, based on the RVE model with 21,340 elements. The results indicate that the elastic modulus of the five RVEs are consistent, while the tensile and compressive strength predictions for the first two models with fewer meshes show significant differences compared to those with more elements. Balancing the impact on result convergence and computational time, this paper ultimately selects the RVE model with 21,340 elements for subsequent numerical studies.

Solid elements were used for fibers and matrices, specifically eight-node hexahedral reduced integration elements (C3D8R) and six-node prismatic elements (C3D6). The interfacial layer employed zero-thickness cohesive elements (COH3D8). To avoid convergence issues due to material damage and failure in finite element calculations, an explicit solution solver, ABAQUS/Explicit, was chosen. Periodic boundary conditions were applied to ensure the continuity of stress and displacement at the boundaries, allowing accurate homogenization of elastic and strength properties with a smaller analysis domain [29,30]. The common approach to introducing periodic boundary conditions in an RVE involves the following: first, creating a periodic RVE; second, meshing it periodically; and finally, defining constraint equations to ensure consistent relative displacements between corresponding nodes on parallel boundary surfaces. Periodic boundary conditions in finite elements are implemented by establishing linear constraint equations at corresponding mesh nodes on parallel opposite faces of the RVE, which are realized through equations in ABAQUS 2017. In the existing RVE, 29,044 equations constrain the relative displacements between corresponding node pairs; six virtual nodes are used to control the six strain components, and these virtual nodes carry the entire mass of the FEM, which is 8.6 × 10^−12^ kg. To accelerate the simulation, the ABAQUS/Explicit solver often requires mass scaling to artificially increase element mass, thus increasing the minimum time increment and reducing total computation time. While this enhances computational efficiency, it introduces additional kinetic energy during quasi-static simulations. Excessive kinetic energy can affect simulation results, so the ratio of total kinetic energy to internal energy should not exceed 5%. To control the impact of mass scaling, a stable time increment of 2.5 × 10^−6^ s was set. The simulation time for the UC model on a computer with an Intel Core i7-12700 (2.1~4.9 GHz) CPU was approximately 15.5 h.

Strain loadings are applied to the RVE through boundary conditions, and the mechanical response of the unidirectional composite is obtained from the homogenized stress $\sigma_{ij}^{0}$ and strain $\varepsilon_{ij}^{0}$ of the RVE. Specifically, the stress field of all elements in the RVE model is obtained through finite element analysis, and then the homogenized stress corresponding to each time step is calculated using a volume averaging method. The formula is as follows:

(1) $$\sigma_{ij}^{0}=\frac{1}{V}\int \sigma_{ij}dV=\frac{1}{V}\sum_{k=1}^{N}\sigma_{ij}^{k}V^{k}$$

where $\sigma_{ij}^{k}$ and $V^{k}$ represent the stress and volume of each element, respectively, while *N* denotes the total number of elements. The average strain $\varepsilon_{ij}^{0}$ can be derived by converting the applied displacement loadings. By calculating the homogenized stress corresponding to different homogenized strains, the stress–strain curve of the UD composite can be obtained, which allows for the prediction of its macroscopic properties.

### 3.2. Material Model

In the RVE model for predicting the transverse properties of composites, it is assumed that carbon fibers will not fail. To simplify calculations, the fibers are assumed to be isotropic linear elastic materials. The material constitutive model is determined by the elastic modulus $E_{f}$ and Poisson’s ratio $\nu_{f}$. According to references [19,31], $E_{f}$ is taken as 20 GPa and $\nu_{f}$ as 0.2.

**Figure 8:**
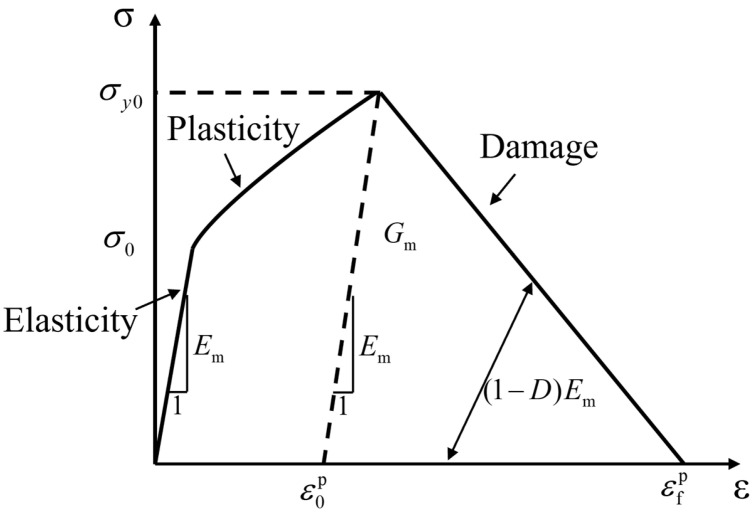
Resin constitutive model curve.

The polymer matrix is considered as an isotropic elastoplastic material, with its constitutive model divided into elastic, plastic, and damage stages, as referenced in [32]. Figure 8 illustrates the constitutive model of polymer matrices, and the following provides descriptions of each of these three stages. The elastic stage of the material’s constitutive model is simulated using the elastic modulus $E_{m}$ and Poisson’s ratio $\nu_{m}$.

The plastic behavior is characterized by the linear Drucker–Prager yield criterion:

(2) $$\left\{\begin{array}{c} F=t-p\tan\beta -d=0\\ t=\frac{1}{2}q\left[1+\frac{1}{k}-(1-\frac{1}{k}){\left(\frac{r}{q}\right)}^{3}\right] \end{array}\right.$$

where *p* represents the hydrostatic stress, *q* represents the von Mises equivalent stress, *r* represents the third invariant of the stress deviator tensor, *β* represents the inclination angle of the linear yield surface in the *p–t* stress plane, *d* represents the cohesion of the material, and *k* represents the ratio of the triaxial tensile yield stress to the triaxial compressive yield stress.

The parameters *β* and *k* used for numerical simulation are determined by the following formulas:

(3) $$\tan\beta =\frac{6\sin\phi }{3-\sin\phi }$$

(4) $$k=\frac{3-\sin\phi }{3+\sin\phi }$$

In Equations (3) and (4), ϕ represents the friction angle of the material according to the Mohr–Coulomb criterion, which can be determined by the yield tensile strength $X_{mt}$ and compressive strength $X_{mc}$ of the resin.

(5) $$\sin\phi =\frac{X_{\mathrm{mc}}-X_{\mathrm{mt}}}{X_{\mathrm{mc}}+X_{\mathrm{mt}}}$$

The tensile and compressive properties of the resin are obtained through resin casting body test experiments, with reference to China’s national standard GB/T 2567-2008 [33]. These tests were conducted at room temperature. For both tensile and compressive tests, five batches were prepared, with each batch consisting of five test specimens. The specific test results are presented in Appendix A. By selecting the average value of the test results in Table A1 and Table A2, the elastic modulus $E_{m}$ = 3.59 GPa, the tensile strength of the polymer resin $X_{mt}=86.6\;MPa$, and the compressive strength $X_{mc}=135.8\;MPa$. The Poisson’s ratio $\nu_{m}$ is taken as 0.35 [19]. According to Equations (3)–(5), the simulated parameters for the resin under the Drucker–Prager criterion are calculated as follows: friction angle β=25.5°, flowstress ratio k=0.86, and the dilation angle is set to zero.

Experimental results [34] indicate that polymer resins exhibit brittle fracture behavior under uniaxial tension, but yield and undergo significant plastic deformation under uniaxial compression and pure shear. Therefore, a ductility criterion is employed to predict damage initiation across different triaxial stress states, assuming that the equivalent plastic strain $\varepsilon_{0}^{P}$ at damage initiation is a function of the stress triaxiality, denoted as η. Under uniaxial tension and compression, η takes values of 1/3 and −1/3, respectively. The corresponding reference equivalent plastic strains at the onset of damage for uniaxial tension and compression, based on [25,34], are set to 0.05 and 0.5, respectively. Damage evolution is related to the damage variable *D*, which increases as damage evolves. The evolution of damage is controlled by a linear softening process, with the ultimate failure strain $\varepsilon_{f}^{P}$ determined by the fracture energy $G_{m}$, which is set to 1 J/m^2^ according to [19]. The previously mentioned model can be implemented in ABAQUS/Explicit, and further details can be found in [35].

**Figure 9:**
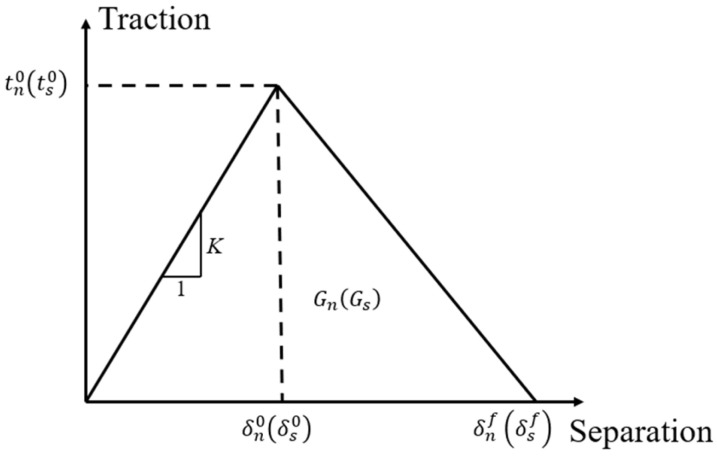
Interface bilinear constitutive model curve.

According to reference [35], the cohesive elements at the simulated interface adopt a bilinear traction–separation constitutive, linking the relative displacement between the top and bottom surfaces of the cohesive elements to the traction vector, as shown in Figure 9. The constitutive model consists of an elastic segment and a damage evolution segment. The stiffness *K* in the elastic segment determines the linear relationship between the normal traction $t_{n}$ and the two shear tractions $t_{s}$, and their corresponding separation displacements $\delta_{n}$ ($\delta_{s}$). The damage initiation displacements $\delta_{n}^{0}$ ($\delta_{s}^{0}$) are determined by the interface normal strength $t_{n}^{0}$ and shear strength $t_{s}^{0}$, along with the quadratic nominal stress criterion, expressed as follows:

(6) $${\left\{\frac{\langle t_{n}\rangle }{t_{n}^{0}}\right\}}^{2}+{\left\{\frac{t_{s}}{t_{s}^{0}}\right\}}^{2}+{\left\{\frac{t_{t}}{t_{t}^{0}}\right\}}^{2}=1$$

where $<t_{n}>$ represents taking zero when $t_{n}$ is negative, which means that normal compressive stress will not cause initial damage to the interface.

The damage evolution stage is a linear softening process, and the Benzeggagh–Kenane (B-K) energy criterion is selected to determine the final damage displacement $\delta_{n}^{f}$ ($\delta_{s}^{f}$). The final damage displacement is determined by the fracture strain energy $G_{n}$ ($G_{s}$), which equals the area under the traction–separation curve. Both the aforementioned constitutive model and failure criterion are implemented using the cohesive element model built into ABAQUS/Explicit.

The interfacial shear strength between carbon fiber and epoxy resin, measured through the microdroplet pull-out test with reference to the experimental method in [36], is 72 MPa. Assuming that the normal strength of the interface between fiber and resin is two-thirds of the interfacial shear strength [20], it is taken as 48 MPa. Due to the lack of experimental data, other properties of the interface element are referenced from values in [20]. In Figure 9, the stiffness *K* of the interface’s linear segment is set to 10^8^ GPa/m, chosen to ensure continuity at the interface between fiber and resin and to avoid altering the stress field around the fiber prior to damage. The mode I fracture energy of the interface, $G_{IC}$, is taken as 2 J/m^2^, while the tangential fracture energies, $G_{IIC}$ and $G_{IIIC}$, are both 100 J/m^2^.

## 4. Results and Discussion

### 4.1. Transverse Tension

**Figure 10:**
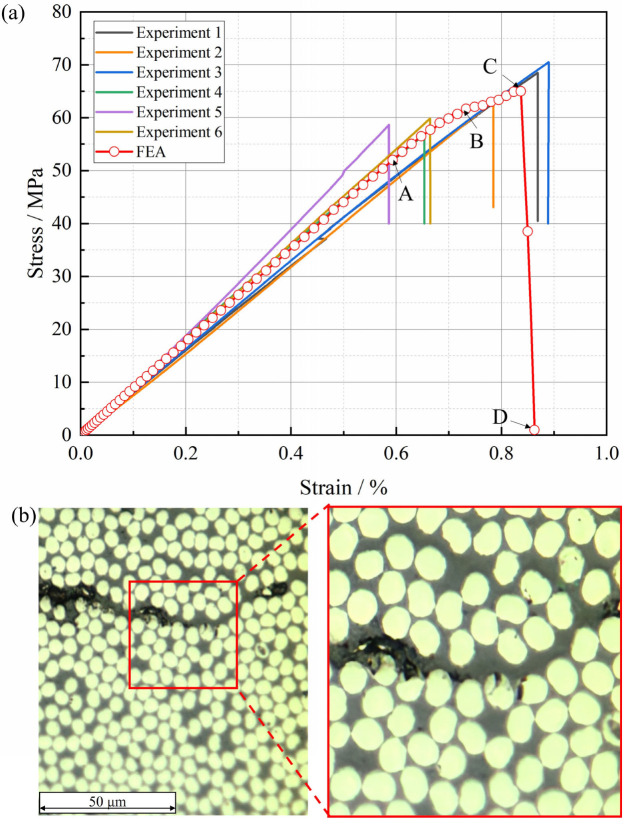
Transverse tensile testing of T800-grade UD CFRP: (**a**) RVE and experimental stress–strain curves; (**b**) microscopic morphology of final failure state in fractography experiments.

The stress–strain curves and failure processes of the T800-grade UD CFRP under transverse tensile loading, both from experiments and the RVE model, are shown in Figure 10. According to Figure 10a, the stress–strain curve of the transverse tensile specimen remains linear until failure, with brittle failure occurring at the maximum load. Table 1 lists the transverse tensile modulus (slope of the curve between 0.1% and 0.3% strain), tensile strength, and failure strain obtained from the curve, with average values of 8.7 GPa, 64 MPa, and 0.74%, respectively. These data are comparable to the levels obtained in tests by Camanho and Lambert [37] for IM7/8552 and by Swanson and Qian [38] for T800/3900-2, indicating the good consistency and high reliability of the experimental test results. The standard deviations (STDVs) and coefficients of variation (CVs) of the tensile properties are also shown in Table 1. The CV for the tensile modulus and tensile strength is 6.9% and 8.1%, respectively, indicating relatively significant dispersion. Possible reasons for this include the inherent anisotropic characteristics and the complexity of the manufacturing process associated with carbon fiber-reinforced composites. Notably, the T800-grade carbon fiber/epoxy resin composites studied in this paper are laboratory-produced materials. Compared to the mature material system IM7/8552 employed by Camanho and Lambert [37], which has CV values of 1.0% and 8.5% for tensile modulus and tensile strength, respectively, although the composites investigated numerically attain comparable performance levels, there is room for further improvement in terms of performance stability.

Figure 10a also displays the stress–strain curve predicted by the RVE model, which exhibits linear behavior consistent with the experimental curve, except for a slight nonlinear trend before ultimate failure. As seen in Table 1, the transverse tensile modulus, ultimate failure stress, and failure strain predicted by FEA are 8.8 GPa, 65.0 MPa, and 0.84%, respectively, differing from the experimental averages by 1.1%, 3.2%, and 13.5%. The elastic modulus and strength results are in good agreement with the experiments, while the failure strain result shows a larger discrepancy due to the nonlinear behavior exhibited by the RVE stress–strain curve before ultimate failure. The fractography observation results in Figure 10b show that the final fracture morphology of the T800-grade CFRP under tensile loading is a crack penetrating through the thickness of the laminates.

**Figure 11:**
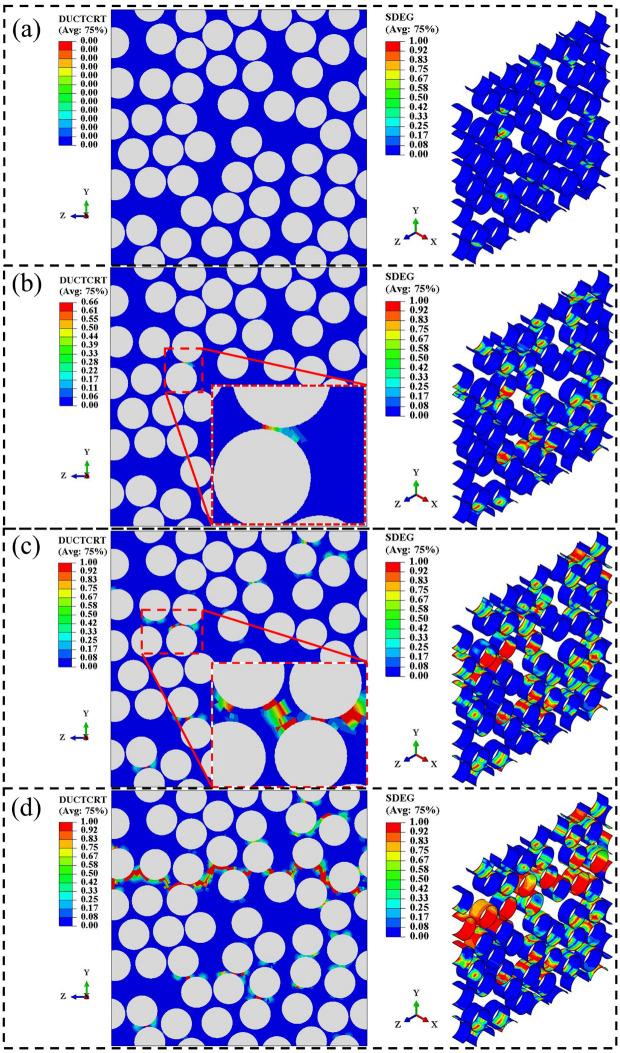
The damage state of elements in the RVE under transverse tensile loading: (**a**) initial damage; (**b**) damage propagation; (**c**) damage at the peak load; (**d**) ultimate failure.

To describe the failure process of the T800-grade CFRP under transverse tensile loading, four characteristic points (A, B, C, and D) were selected on the stress–strain curve predicted by the RVE model in Figure 10a. These four points represent four distinct damage states: initial damage, damage propagation, damage at peak load, and final failure, respectively. These corresponding element damage states are shown in Figure 11, which employs two state variables. “SDEG” represents the damage state of each element, where a value greater than zero indicates that the element is damaged, and a value equal to one indicates that the element is completely damaged. “DUCTCRT” specifically refers to the damage state of the resin elements, where a value greater than zero indicates damage to the element, and a value equal to one indicates complete damage to the element. At point A on the curve, damaged interface elements first appeared under transverse (Y-direction) tensile loading, as shown in Figure 11a. At this stage, no resin element damage occurred, and the curve slope slightly decreased due to interface elements’ damage. As the strain load increased, more interface elements sustained damage, and simultaneously, resin elements near the interface elements entered a damaged state due to stress concentration, as shown in Figure 11b. At this point, the overall stiffness of the model significantly decreased due to the degradation of numerous interface elements and some resin elements, corresponding to point B on the curve. Subsequently, the curve reached point C in a nonlinear state, where the RVE model’s load-bearing capacity peaked. At this stage, some elements in resin-poor areas between fibers experienced complete failure, as shown in Figure 11c. Finally, the curve descends to point D, where extensive damage to interface and resin elements resulted in through-cracks perpendicular to the loading direction (Y-direction), as shown in Figure 11d. This phenomenon is consistent with the fractography experimental observations shown in Figure 11b.

### 4.2. Transverse Compression

**Figure 12:**
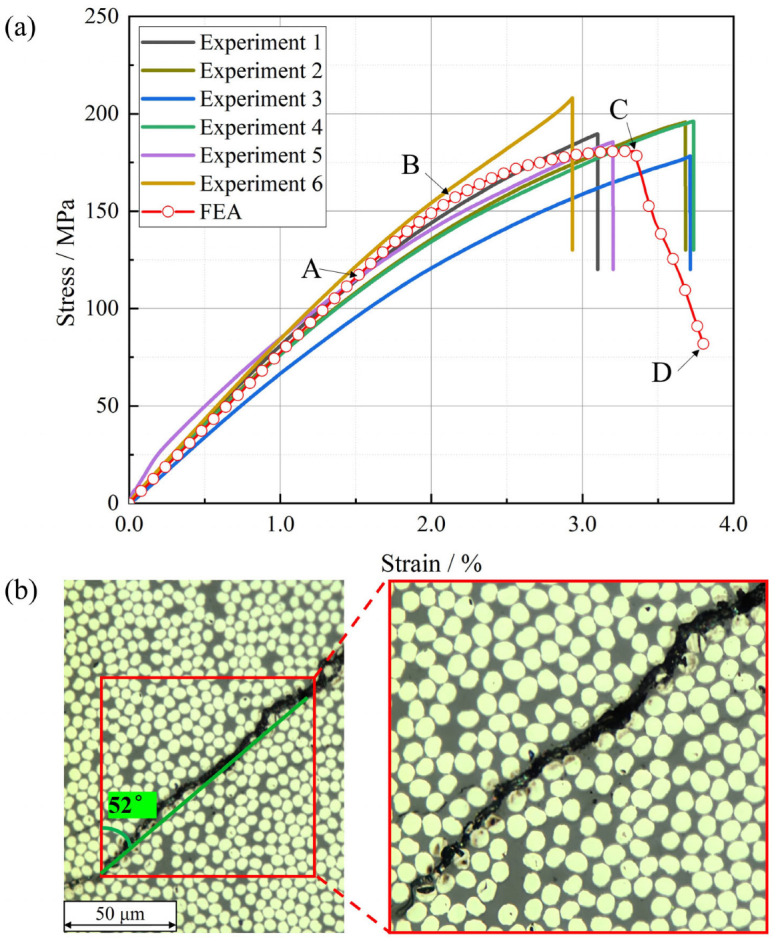
Transverse compressive testing of the T800-grade UD CFRP: (**a**) RVE and experimental stress–strain curves; (**b**) microscopic morphology of final failure state in fractography experiments.

The stress–strain curves and failure process of the T800-grade CFRP under transverse compressive loading, along with the corresponding RVE model predictions, are illustrated in Figure 12. Unlike under tensile loading, the stress–strain curve of the specimen under compressive loading exhibits significant nonlinear characteristics, with a continuously decreasing slope until ultimate failure. The compressive modulus (slope of the curve between strains of 0.1% and 0.3%), compressive strength, and failure strain of the specimens are summarized in Table 2, with mean values of 8.4 GPa, 197.1 MPa, and 3.43%, respectively, demonstrating good comparability to the test results of other similar materials reported in [37,38]. Table 2 also lists the STDV and CV for elastic modulus and strength. Specifically, the CV values for compressive modulus and strength are 11.3% and 4.8%, respectively, demonstrating a significant degree of dispersion similar to that observed in tensile properties. Compared to the values of 1.0% and 10.2% reported in [37], there is considerable room for improvement in terms of performance stability.

The stress–strain curve predicted by the RVE model aligns well with the experimental curve. The predicted transverse compressive modulus is 8.7 GPa, the ultimate failure stress is 180.3 MPa, and the failure strain is 3.3%. These predictions deviate by 3.6%, −8.5%, and −3.8% from the experimental mean values, respectively, validating the accuracy of the model.

**Figure 13:**
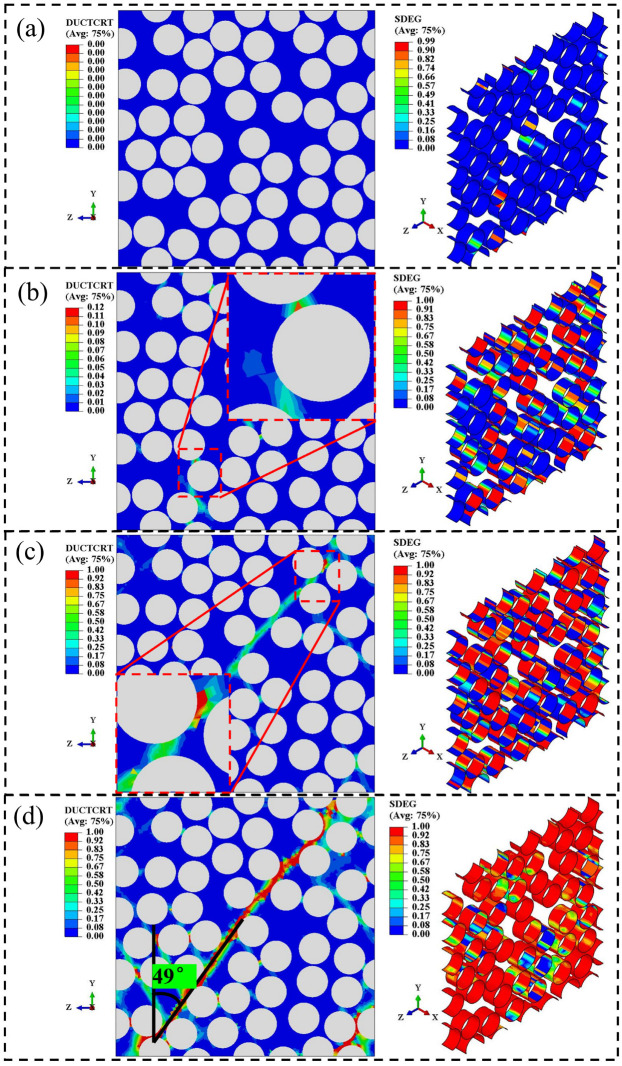
The damage state of elements in the RVE under transverse compressive loading: (**a**) initial damage; (**b**) damage propagation; (**c**) damage at the peak load; (**d**) ultimate failure.

Similar to the tensile curve, four characteristic points (labeled A, B, C, and D) are also selected on the compressive stress–strain curve predicted by the RVE model in Figure 12a, representing damage initiation, damage propagation, damage at peak load, and ultimate damage, respectively, to describe the transverse compressive failure process. The failure states of the elements within the RVE are depicted in Figure 13. At point A, interfacial elements within the RVE model begin to sustain damage, as shown in Figure 13a, while no resin elements are damaged at this stage. Subsequently, as the load reaches point B, a significant number of damaged interfacial elements cause stress concentration, leading to damage in nearby resin elements, as illustrated in Figure 13b. At point C, the curve reaches its peak. Figure 13c shows that damaged resin elements form an oblique line within the model at this point. Unlike in tension, the resin exhibits more pronounced plastic characteristics under compressive loading, resulting in many resin elements being damaged but not yet failing, with corresponding damage factors still less than one. Consequently, the global stiffness of the model decreases significantly before ultimate failure, and the compressive stress–strain curve exhibits more obvious nonlinear characteristics compared to the tensile curve. Finally, at point D on the curve, the model is completely destroyed and cannot bear any load, with damaged resin elements forming a crack at a 49° angle to the positive Y-direction, as shown in Figure 13d. The predicted final failure morphology is consistent with the observations from fractography experiments (as shown in Figure 12b), with a −5.8% deviation compared to the actual failure angle of 52°, indicating the good accuracy of the model. The angle of cracks calculated by the model is related to the resin’s yield failure criterion, which can lead to discrepancies between the model-calculated crack angle and actual results. Similar discrepancies have also been observed in other studies, such as [15,19].

Comparison of the transverse tensile and compressive test results for the T800-grade UD CFRP reveals that the elastic moduli of the two are similar. However, significant differences arise in failure strength and failure strain due to distinct failure modes of the resin and interface under tensile and compressive states. Specifically, under tensile loading, the interface primarily bears normal tensile forces, leading to normal-dominated damage and failure of cohesive elements in the RVE model. The resin exhibits brittle failure characteristics in this state. Conversely, under compressive loading, the interface mainly withstands shear loads, corresponding to shear-dominated damage and failure of cohesive elements in the RVE model, while the resin exhibits noticeable elastoplastic behavior. These differences manifest in the stress–strain curves, where the tensile curve shows a distinct linear characteristic, whereas the compressive curve exhibits a pronounced nonlinear feature. Similar findings have been reported in the literature [14,19,39]. Microscopic failure morphologies also support these observations. Combining finite element analysis results, it is found that interface elements aligned with the tensile loading direction undergo initial separation and failure, causing stress concentration in surrounding resin elements, which then fail due to their brittle nature, ultimately linking up to form a through-thickness crack perpendicular to the loading direction. Under compressive loading, interface and resin elements distributed at certain angles fail due to shear stresses, eventually connecting to form a crack at an angle determined by the internal friction angle, aligning with the results presented in [16,19]. Through a combination of experimental testing, finite element simulation in micromechanics, and microscopic damage observation, various methods have been employed to explore the failure mechanisms of the T800-grade UD CFRP under both transverse tensile and compressive loads. These findings provide beneficial perspectives for the engineering applications of this material.

## 5. Conclusions

This paper aims to investigate the transverse tensile and compressive properties and underlying failure mechanisms of a T800-grade UD CFRP through experimental and micromechanical FEMs. The following key findings were obtained:

1. The transverse tensile modulus of the T800-grade material is 8.7 GPa, with a tensile strength of 64 MPa and a failure strain of 0.74%. Experimental results indicate that the stress–strain curve exhibits linear characteristics until final failure. The micromechanical FEM model predicts a stress–strain curve that linearly matches the experimental curve at the initial stage but exhibits some nonlinearity before ultimate failure. Simulations of the failure process reveal that interface elements first fail under transverse tensile loading, subsequently leading to damage in nearby resin elements due to stress concentration. Due to the brittleness of the resin under tension, there is no significant plastic deformation in the overall structure before final failure, explaining the linear characteristic of the experimentally tested stress–strain curve. The failed interface elements are predominantly dominated by normal failure, combined with the brittle nature of the resin, resulting in failed elements perpendicular to the loading direction connecting to form through-thickness cracks, which is consistent with fracture observations. The predicted values for transverse tensile modulus, tensile strength, and failure strain are close to the experimental results, with deviations of −1.1%, 3.2%, and 13.5%, respectively.
2. For the transverse compressive properties of the T800-grade material, the modulus is 8.4 GPa, with a compressive strength of 197.1 MPa and a failure strain of 3.43%. During testing, the experimental stress–strain curve exhibits noticeable nonlinearity before failure. The micromechanical FEM model accurately predicts a similar trend. Simulation results show that under transverse compression, interface elements at an angle to the loading direction first fail, subsequently initiating damage in adjacent resin elements. However, the plastic nature of the resin under compression slows down the failure of the overall structure, leading to a gradual reduction in stiffness until final failure, which corresponds to the nonlinear characteristic of the experimental curve. The failed interface elements concentrate along an oblique line at an angle to the loading direction, related to the internal friction angle of the plastic criterion for resin elements. The ultimately failed resin and interface elements connect to form cracks oriented at an angle of 49° to the loading direction, which is very close to the 52° angle observed in fracture testing. The predicted transverse compressive modulus, compressive strength, and failure strain are in good agreement with the experimental results, with differences of 3.6%, −8.5%, and −3.8%, respectively.

In summary, this paper utilizes experimental testing, fractography observation, and micromechanical FEM to obtain the transverse tensile and compressive mechanical properties of a T800-grade UD CFRP. It explains the mechanical characteristics of linearity in tension and nonlinearity in compression due to different failure modes, as well as the reasons for the formation of various fracture features. These findings contribute to a comprehensive understanding of the transverse mechanical properties of this T800-grade carbon fiber–epoxy composite system, providing a basis for its future applications in high-performance-demanding scenarios such as in the aviation or aerospace industry.

## Figures and Tables

**Table 1:** Experimental and numerical results for transverse tensile properties of the T800-grade CFRP unidirectional composite material.

No.	Tensile Modulus/GPa	Tensile Strength/MPa	Failure Strain
1	8.1	68.8	0.87%
2	8.0	63.3	0.79%
3	8.3	70.5	0.89%
4	8.9	56.8	0.65%
5	9.7	58.7	0.59%
6	9.0	59.9	0.66%
Mean value	8.7	63.0	0.74%
Standard deviation	0.6	5.1	0.1%
Coefficient of variation	6.9%	8.1%	15.5%
FEA	8.8	65.0	0.84%
Model accuracy	1.1%	3.2%	13.5%

Note: Model accuracy is calculated by $\frac{(\mathrm{FEA}-\mathrm{Mean}\;\mathrm{value})}{\mathrm{Mean}\;\mathrm{value}}\times 100\%$.

**Table 2:** Experimental and numerical results for transverse compressive properties of the T800-grade CFRP unidirectional composite material.

No.	Compressive Modulus/GPa	Compressive Strength/MPa	Failure Strain
1	9.0	190.0	3.09%
2	8.0	196.7	3.69%
3	6.9	190.7	3.81%
4	8.1	210.1	3.75%
5	10.0	186.0	3.30%
6	8.4	209.1	2.94%
Mean value	8.4	197.1	3.43%
Standard deviation	1.0	9.4	0.3%
Coefficient of variation	11.3%	4.8%	9.9%
FEA	8.7	180.3	3.3%
Model accuracy	3.6%	−8.5%	−3.8%

Note: Model accuracy is calculated by $\frac{(\mathrm{FEA}-\mathrm{Mean}\;\mathrm{value})}{\mathrm{Mean}\;\mathrm{value}}\times 100\%$.

## Data Availability

The original contributions presented in this study are included in the article. Further inquiries can be directed to the corresponding authors.

## Abbreviations

The following abbreviations are used in this manuscript:

UD	Unidirectional
CFRP	Carbon fiber-reinforced polymer
RVE	Representative volume element
FEM	Finite element method

## Appendix A

**Table A1:** Tensile test results of resin casting body.

Batch No.	Spec. No.	Width/mm	Thickness/mm	Tensile Modulus/GPa	Tensile Strength/MPa	Elongation at Break/%
1	1	5.15	2.44	3.45	89.8	3.88
	2	5.18	2.49	3.38	86.6	3.67
	3	5.18	2.55	3.42	69.9	2.50
	4	5.18	3.09	3.32	95.6	4.28
	5	5.20	2.71	3.32	87.9	3.84
2	1	5.18	2.92	3.45	87.8	3.66
	2	5.19	2.55	3.43	65.6	2.57
	3	5.17	2.66	3.55	87.6	3.73
	4	5.21	2.55	3.59	98.4	4.39
	5	5.28	3.01	3.64	81.0	3.19
3	1	5.10	3.08	3.64	97.1	3.81
	2	5.11	3.20	3.50	92.8	3.65
	3	5.09	3.14	3.55	102.0	4.47
	4	5.11	3.19	3.77	82.8	3.06
	5	5.11	3.11	3.72	97.0	3.95
4	1	5.14	2.93	3.16	83.4	3.48
	2	5.07	2.96	4.11	84.3	3.59
	3	5.14	2.98	3.65	82.9	3.36
	4	5.16	2.99	3.66	86.9	3.79
	5	5.21	2.92	3.73	82.5	3.31
5	1	5.20	2.99	3.63	83.4	3.39
	2	5.22	2.90	3.68	84.0	3.53
	3	5.27	2.91	3.62	82.5	3.40
	4	5.30	2.93	3.66	85.5	3.59
	5	5.22	2.92	3.75	87.1	3.75

**Table A2:** Compressive test results of resin casting body.

Batch No.	Specimen No.	Width/mm	Thickness/mm	Compressive Modulus/GPa	Compressive Strength/MPa
1	1	10.05	8.99	3.54	134
	2	10.03	8.81	3.61	134
	3	10.02	8.92	3.76	133
	4	10.03	8.90	3.48	131
	5	10.04	8.92	3.58	138
2	1	10.09	8.96	3.54	132
	2	10.02	8.82	3.60	133
	3	10.02	8.86	3.77	139
	4	10.05	8.90	3.60	135
	5	10.07	8.86	3.67	130
3	1	10.13	8.93	3.58	139
	2	10.12	8.97	3.62	139
	3	10.18	8.99	3.62	129
	4	10.06	8.93	3.56	134
	5	10.09	8.97	3.62	137
4	1	10.03	8.91	3.60	138
	2	10.05	8.96	3.55	136
	3	9.93	8.84	3.58	136
	4	10.08	9.04	3.64	140
	5	10.18	8.93	3.59	139
5	1	10.11	8.87	3.64	139
	2	10.12	9.02	3.56	135
	3	10.05	8.97	3.69	137
	4	10.11	8.95	3.60	135
	5	10.07	8.94	3.58	139
